# Vitamin D deficiency in a subfertility population and the impact of COVID‐19 lockdowns

**DOI:** 10.1002/ijgo.70124

**Published:** 2025-04-07

**Authors:** Catherine M. Windrim, Daniel Kane, Grainne Kelleher, Edgar Mocanu

**Affiliations:** ^1^ Obstetrics and Gynecology Rotunda Hospital Dublin Ireland; ^2^ Royal College of Surgeons Ireland Unit Rotunda Hospital Dublin Ireland; ^3^ Department of Laboratory Medicine Rotunda Hospital Dublin Ireland

**Keywords:** female, fertility, patient education, pregnancy complications, vitamin D deficiency

## Abstract

**Objective:**

To evaluate the burden of vitamin D deficiency in female patients attending a fertility clinic in a tertiary referral center, assess temporal trends—including the potential impact of COVID‐19 lockdowns—and explore socioeconomic disparities in vitamin D levels.

**Methods:**

This retrospective cohort study analyzed vitamin D measurements from 765 female patients (mean age 35.7 ± 5.8 years) attending a fertility clinic between March 2010 and May 2022. Vitamin D status was categorized as deficient (<30 nmol/L), insufficient (30–50 nmol/L), or normal (>50 nmol/L). Comparative analyses examined pre‐ and post‐COVID periods and healthcare funding status.

**Results:**

Overall, 39.9% (*n* = 305) of patients exhibited suboptimal vitamin D levels, with 8.8% (*n* = 67) deficient and 31.1% (*n* = 238) insufficient. Mean serum 25(OH)D was 62.8 ± 27.4 nmol/L. No statistically significant difference was observed between pre‐COVID (44.2% suboptimal) and post‐COVID (38.1% suboptimal) periods (OR 0.78, 95% CI: 0.57–1.06, *P* = 0.110). However, marked seasonal variation was identified, with winter values significantly lower than summer values (45.3 ± 24.6 vs. 72.1 ± 28.3 nmol/L, *P* < 0.001). Furthermore, state‐funded patients had a significantly higher rate of suboptimal vitamin D status (50.2%, *n* = 107) compared to self‐funded patients (35.9%, *n* = 198, *P* < 0.001).

**Conclusion:**

Our findings demonstrate a high prevalence of suboptimal vitamin D levels in a subfertility population, with significant seasonal fluctuations and notable socioeconomic disparities. Despite initial concerns, COVID‐19 lockdown measures did not appear to adversely affect overall vitamin D status. These results support the incorporation of routine vitamin D screening in infertility evaluations and the implementation of targeted supplementation, particularly in economically vulnerable groups and during winter months.

## 1 INTRODUCTION

Vitamin D has emerged as a critical factor in reproductive health, influencing both fertility and pregnancy outcomes. Beyond its well‐known role in calcium metabolism and bone health, vitamin D is now recognized as a key regulator of ovarian function, implantation, and pregnancy maintenance.^1^, ^2^, ^3^, ^4^, ^5^, ^6^ Vitamin D receptors (VDRs) are expressed in reproductive tissues, including the ovaries, endometrium, and placenta, suggesting a direct role in female fertility and pregnancy.^3^, ^5^, ^7^, ^8^, ^9^

During pregnancy, maternal vitamin D in its active form, 1,25‐dihydroxyvitamin D3 or calcitriol, levels rise 2–3 times above non‐pregnant levels, driven by increased activity of 1α‐hydroxylase mostly within the maternal kidney but also placental activation.^3^ Vitamin D deficiency disrupts this balance and has been implicated in adverse pregnancy outcomes, including pre‐eclampsia, spontaneous preterm birth, and fetal growth restriction.^3^ Importantly, maternal vitamin D status directly influences fetal vitamin D stores, as 25‐hydroxyvitamin D, 25(OH)D or calcifediol, freely crosses the placenta and serves as the primary source for the developing fetus.^1^, ^3^

A growing body of literature highlights the impact of vitamin D status on reproductive outcomes. Recent systematic reviews and meta‐analyses have shown that vitamin D supplementation significantly improves clinical pregnancy rates among infertile women undergoing assisted reproductive technologies (ART) and reduces adverse pregnancy outcomes. For instance, Meng et al. found that vitamin D supplementation improved clinical pregnancy rates among infertile women undergoing ART with an odds ratio of 1.70 (95% confidence interval [CI]: 1.24–2.34, *P* = 0.001).^2^ Additionally, vitamin D deficiency (<50 nmol/L) is associated with a significantly increased risk of preterm birth (odds ratio [OR] = 1.28, 95% CI: 1.08–1.52), gestational diabetes (OR = 1.38, 95% CI: 1.22–1.57), small‐for‐gestational‐age infants (OR = 1.43, 95% CI: 1.08–1.91), and recurrent miscarriage (OR = 4.02, 95% CI: 2.23–7.25).^5^ Conversely, vitamin D supplementation (>400 IU/day) has been linked to a reduction in pre‐eclampsia (OR = 0.29, 95% CI: 0.09–0.95), miscarriage (OR = 1.60, 95% CI: 1.11–2.30), and fetal or neonatal mortality (RR = 0.69, 95% CI: 0.482–0.985).^5^ These findings underscore the importance of maintaining adequate vitamin D levels for improving both fertility and perinatal outcomes.

In addition, a systematic review and meta‐analysis by Guang et al. found that vitamin D supplementation during pregnancy significantly reduced the risk of small‐for‐gestational‐age (SGA) infants (RR = 0.72, 95% CI: 0.52–0.99) and increased birth weight by an average of 75.38 g (95% CI: 22.88–127.88 g).^1^ Moreover, neonates whose mothers received prenatal vitamin D supplementation had higher 25(OH)D levels (MD = 13.50 ng/mL, 95% CI: 10.12–16.87 ng/mL), underscoring the essential role of maternal vitamin D sufficiency in optimizing fetal growth.^1^ Notably, vitamin D supplementation at doses ≤2000 IU/day was associated with a 65% reduction in fetal or neonatal mortality (RR = 0.35, 95% CI: 0.15–0.80), further supporting the need for adequate vitamin D intake during pregnancy.^1^

Given these findings, our study aimed to assess the prevalence of vitamin D insufficiency and deficiency in patients presenting to a tertiary fertility clinic. We conducted a retrospective cohort analysis examining temporal trends in vitamin D status, with a particular focus on differences before and after the COVID‐19 pandemic, variations by season and age group, and disparities based on payer status (self‐funded vs. state‐funded).

## 2 MATERIALS AND METHODS

### 2.1 Study design

We conducted a retrospective cohort analysis of serum vitamin D (25[OH]D) measurements from female patients attending the Rotunda Hospital fertility clinic between March 2010 and May 2022. Inclusion criteria encompassed all patients with recorded vitamin D levels during this period. Because the study was retrospective and included all available measurements, a formal prospective sample size calculation was not performed; instead, our analysis represents the entire cohort of eligible patients.

### 2.2 Data collection and processing

Data was obtained from the laboratory information system at the Rotunda Hospital Laboratory. The dataset contained anonymized serum vitamin D measurements (25[OH]D) in nmol/L with corresponding sampling dates, patient age at time of sampling, and healthcare funding classification (state‐funded vs. self‐funded care pathways). The temporal analysis spanned from March 2010 to May 2022, with March 13, 2020, designated as the division between pre‐ and post‐COVID‐19 periods, corresponding to the implementation of initial lockdown measures in Ireland. The laboratory system's existing anonymization protocols ensured data protection compliance throughout the analysis period.

### 2.3 Measurement of vitamin D concentrations

All serum vitamin D (25[OH]D) measurements were performed at the Rotunda Hospital, Dublin laboratory utilizing standardized vitamin D assay protocols on the e402 module of the Roche Cobas Pure analyzer. This ensured methodologic consistency across the entire study period.

### 2.4 Vitamin D status categorization

Institutional protocols defined vitamin D status according to established threshold values. Deficiency was classified as measurements below 30 nmol/L, insufficiency as measurements between 30 and 50 nmol/L, and normal status as measurements above 50 nmol/L. For analytical purposes, deficient and insufficient categories were consolidated to define suboptimal vitamin D status as any measurement below 50 nmol/L, particularly for temporal comparisons spanning the COVID‐19 period.

### 2.5 Statistical analysis

Statistical analysis was performed using SPSS software package (version 26; SPSS, Chicago, Illinois). The analytical framework comprised several sequential components. Primary analysis evaluated distributional characteristics of vitamin D levels, including mean, median, and standard deviation calculations. Temporal comparison of pre‐ and post‐COVID‐19 periods employed Chi‐square testing for categorical variables and student's *t*‐test for continuous measures. Healthcare funding stratification analysis utilized similar methodologic approaches to assess vitamin D status distribution between state‐funded and self‐funded cohorts.

Secondary analyses encompassed age‐stratified assessment of vitamin D status and seasonal variation analysis comparing winter (December–February) versus summer (June–August) measurements. The analysis included calculation of odds ratios with 95% confidence intervals for suboptimal vitamin D status across healthcare funding categories. Statistical significance was uniformly defined as a *P* value less than 0.05. Results are presented with appropriate precision metrics including confidence intervals, where applicable.

### 2.6 Ethical considerations

Ethical approval was granted by the Rotunda Hospital Research Ethics Committee (RAG‐2024‐011). As a retrospective study utilizing routinely collected anonymized data, individual patient consent was not required. All procedures adhered to European GDPR standards.

## 3 RESULTS

### 3.1 Overall vitamin D status

The study included 765 female patients with a mean age of 35.7 ± 5.8 years. The overall mean serum vitamin D level was 62.8 ± 27.4 nmol/L (median 59.9 nmol/L). Suboptimal vitamin D status was observed in 39.9% (*n* = 305) of patients, with 8.8% (*n* = 67) classified as deficient and 31.1% (*n* = 238) as insufficient (Table A1).

### 3.2 Temporal analysis: Pre‐ versus post‐COVID‐19

The dataset comprised 231 measurements from the pre‐COVID period (30.2%) and 534 measurements from the post‐COVID period (69.8%). There was no statistically significant difference in vitamin D levels between pre‐COVID (mean 61.4 ± 27.2 nmol/L) and post‐COVID periods (mean 63.5 ± 27.8 nmol/L; *P* = 0.324). Moreover, the proportion of suboptimal vitamin D status was similar between periods, with 39.4% (*n* = 92) pre‐COVID and 40.1% (*n* = 214) post‐COVID patients affected (*P* = 0.870) (Table A2).

### 3.3 Seasonal variation

Seasonal analysis revealed significant differences in vitamin D levels. Mean serum 25(OH)D during winter months was 45.3 ± 24.6 nmol/L compared to 72.1 ± 28.3 nmol/L during summer months (*P* < 0.001), reflecting the expected impact of sunlight exposure (Table A3).

### 3.4 Healthcare funding and socioeconomic disparities

A significant disparity in vitamin D status was observed between healthcare funding groups. Private (self‐funded) patients (50.2%, *n* = 552) had a higher mean vitamin D level (64.7 ± 27.0 nmol/L) compared with state‐funded patients (35.9%, *n* = 213; mean 56.2 ± 29.2 nmol/L, *P* < 0.001). Suboptimal vitamin D status was significantly more prevalent among state‐funded patients compared to self‐funded patients (OR 1.81, 95% CI: 1.32–2.48, *P* < 0.001).

### 3.5 Age‐stratified analysis

Further analysis demonstrated that the 40–45 year age group exhibited a significant improvement in vitamin D status post‐COVID, with suboptimal rates decreasing from 55.0% to 26.9% (*P* = 0.001) after adjusting for seasonality and funding status.

## 4 DISCUSSION

Vitamin D deficiency is a well‐documented public health issue, particularly among women of reproductive age. Our study yielded several clinically significant findings with important implications for reproductive healthcare delivery. The observed 39.87% prevalence of suboptimal vitamin D levels among fertility clinic attendees aligns with established epidemiologic patterns documented in comparable populations. Our investigation extends previous research by O'Riordan et al. and McGowan et al. regarding vitamin D deficiency in the Irish obstetric population, with our findings demonstrating similar patterns specifically in preconception patients.^10^, ^11^ This establishes a critical window for therapeutic intervention prior to pregnancy establishment, potentially mitigating obstetrical complications targeted supplementation.

Our analysis revealed a striking disparity in vitamin D status between healthcare funding pathways, with state‐funded patients demonstrating substantially higher rates of suboptimal levels (50.23%) compared to self‐funded patients (35.87%, *P* < 0.001). This socioeconomic gradient in nutritional status suggests underlying determinants of vitamin D deficiency that transcend purely physiological mechanisms. These disparities align with broader socioeconomic patterns documented by Dong et al. who identified socioeconomic factors as important mediators of psychological health and relationship quality among infertility patients during the pandemic.^12^ Implementation of accessible supplementation programs could potentially optimize reproductive outcomes in socioeconomically vulnerable populations.

Contrary to initial hypotheses, our temporal analysis demonstrated remarkable stability in vitamin D status throughout the pandemic period. The absence of significant variation in vitamin D deficiency rates before and after pandemic‐related restrictions presents an intriguing epidemiologic finding, aligning with observations from Verona, Italy, where similar stability in vitamin D levels was documented throughout the lockdown period.^13^ This unexpected maintenance of vitamin D levels may be attributed to increased opportunity for outdoor activity during work‐from‐home protocols, coinciding with favorable weather conditions during Ireland's lockdown period.

This stability in vitamin D levels contrasts with other research on COVID‐19's impact on women's health. La Verde et al. observed significant increases in gestational diabetes mellitus rates during lockdown (9.3% vs. 3.4%, *P* < 0.001), attributed to higher pregnancy weight gain and increased body mass index (BMI, calculated as weight in kilograms divided by the square of height in meters). This discrepancy between maintained vitamin D status but worsened metabolic parameters suggests differential impacts of pandemic restrictions on various physiological systems. As Almeida et al. documented in their comprehensive review of COVID‐19's impact on women's mental health, pandemic‐related stressors have particularly affected women through multiple pathways, including increased domestic responsibilities, childcare burdens, and limited access to reproductive healthcare services, which may indirectly influence nutritional status through behavioral and lifestyle changes.^14^ Additionally, Adewole and Omotoso found that females requiring reproductive services faced disproportionate access challenges during lockdown compared to males.^15^ These findings collectively emphasize the need for targeted interventions for women's reproductive health during public health emergencies.

Of particular relevance to our study is the relationship between vitamin D receptor expression and pregnancy outcomes in the context of COVID‐19. Condac et al. recently demonstrated that COVID‐19‐positive pregnant women exhibited lower placental VDR levels compared to vaccinated and unvaccinated controls, correlating with reduced birth weights and gestational age at delivery. These findings suggest an intrinsic relationship between vitamin D signaling, COVID‐19 and pregnancy outcomes, emphasizing the importance of addressing vitamin D status as part of comprehensive reproductive healthcare.

Contemporary global trends indicate increasing prevalence of vitamin D deficiency despite enhanced understanding of its physiological significance. Several jurisdictions have implemented systematic interventions. For example, Nordic countries have mandated fortification of dairy products and achieved substantial reductions in deficiency rates despite geographical challenges.^16^ Similar public health strategies merit consideration in the Irish context, particularly given the documented socioeconomic and seasonal disparities.

Several methodologic limitations warrant acknowledgment. The absence of data regarding routine vitamin D supplementation practices among participants constrains interpretation of observed patterns. Additionally, formal socioeconomic stratification beyond healthcare funding status was not performed. Nevertheless, our findings clearly demonstrate substantial vitamin D deficiency prevalence in this fertility clinic population, where targeted supplementation could potentially enhance reproductive outcomes and optimize maternal‐fetal health.

This present study demonstrates the critical importance of incorporating vitamin D assessment into standard infertility evaluation protocols. The documented prevalence of suboptimal vitamin D status, particularly among state‐funded patients, supports implementation of systematic screening and supplementation strategies. This approach represents an opportunity for preventive care in the preconception period. Such intervention could potentially reduce fertility treatment burden and subsequent obstetric complications. Additionally, it addresses persistent socioeconomic disparities requiring targeted public health intervention.

## 5 CONCLUSION

Our findings support three key recommendations: First, we recommend the recognition of infertile patients as a population particularly suitable for preventive intervention prior to pregnancy establishment. Second, the incorporation of vitamin D measurement into routine infertility investigations, given that more than one‐third of patients demonstrate suboptimal levels. Third, the implementation of systematic vitamin D supplementation protocols, particularly in winter month, to optimize reproductive health and mitigate pregnancy‐associated risks. These evidence‐based recommendations provide a framework for enhancing fertility care protocols and improving patient outcomes.

## AUTHOR CONTRIBUTIONS

All authors contributed to the acquisition of data, analysis and interpretation of data, manuscript writing, and final approval of the manuscript.

## CONFLICT OF INTEREST STATEMENT

The authors have no conflicts of interest to declare.

## Data Availability

The data that support the findings of this study are available from the corresponding author upon reasonable request, subject to appropriate data protection and ethical considerations.

## APPENDIX A

See Tables A1–A3.A1, A2, A3

**TABLE A1:** Baseline characteristics and overall vitamin D distribution.

Characteristic	Value	95% CI
Mean age, years (SD)	35.7 (5.8)	35.3–36.1
Mean vitamin D, nmol/L (SD)	62.8 (27.4)	60.9–64.7
Median vitamin D, nmol/L (IQR)	59.9 (45.2–81.3)	57.8–62.0
Status classification, *n* (%)		
Normal (>50 nmol/L)	460 (60.13)	56.6–63.6
Insufficient (30–50 nmol/L)	238 (31.11)	27.9–34.5
Deficient (<30 nmol/L)	67 (8.76)	6.9–11.0

Abbreviations: CI, confidence interval; IQR, interquartile range; SD, standard deviation.

**TABLE A2:** Comprehensive stratification of vitamin D status by healthcare setting and COVID‐19 period (*n* = 765).

Parameter	Pre‐COVID period	Post‐COVID period	Total	*P* value
Total patients, *n* (%)	231 (30.2)	534 (69.8)	765 (100.0)	—
Mean age, years (SD)	35.8 (5.9)	35.6 (5.7)	35.7 (5.8)	0.664
Mean vitamin D levels, nmol/L (SD)	61.4 (27.2)	63.5 (27.8)	62.8 (27.4)	0.324
Healthcare setting				
Private care, *n* (%)	179 (77.5)	373 (69.9)	552 (72.2)	0.030
Public care, *n* (%)	52 (22.5)	161 (30.1)	213 (27.8)	
Vitamin D status				
Private care	*n* = 179	*n* = 373	*n* = 552	
Normal (>50 nmol/L), *n* (%)	114 (63.7)	240 (64.3)	354 (64.1)	0.935
Insufficient (30–50 nmol/L), *n* (%)	52 (29.1)	109 (29.2)	161 (29.2)	
Deficient (<30 nmol/L), *n* (%)	13 (7.2)	24 (6.5)	37 (6.7)	
Mean level, nmol/L (SD)	63.5 (26.8)	65.2 (27.1)	64.7 (27.0)	0.488
Public care	*n* = 52	*n* = 161	*n* = 213	
Normal (>50 nmol/L), *n* (%)	26 (50.0)	80 (49.7)	106 (49.8)	0.691
Insufficient (30–50 nmol/L), *n* (%)	17 (32.7)	60 (37.3)	77 (36.2)	
Deficient (<30 nmol/L), *n* (%)	9 (17.3)	21 (13.0)	30 (14.0)	
Mean level, nmol/L (SD)	54.2 (28.9)	56.8 (29.4)	56.2 (29.2)	0.576

*Note*: Statistical analysis: Student's *t*‐test for continuous variables; Chi‐square test for categorical variables.

Abbreviation: SD, standard deviation.

**TABLE A3:** Combined temporal and healthcare setting analysis of vitamin D status.

Parameter	Pre‐COVID	Post‐COVID	*P* value
Overall analysis			
Number of patients	231	534	—
Mean vitamin D, nmol/L (SD)	61.4 (27.2)	63.5 (27.8)	0.324
Seasonal variation			
Winter mean, nmol/L (SD)	44.8 (23.9)	45.6 (25.1)	0.867
Summer mean, nmol/L (SD)	71.3 (27.8)	72.6 (28.7)	0.743
Healthcare setting analysis			
Private care suboptimal, % (*n*)	36.3 (65)	35.7 (133)	0.891
Public care suboptimal, % (*n*)	50.0 (26)	50.3 (81)	0.969
Private vs. public OR (95% CI)	1.0 (ref)	1.81 (1.32–2.48)	<0.001

*Note*: Suboptimal defined as vitamin D <50 nmol/L. Statistical analysis: Student's *t*‐test for continuous variables; Chi‐square test for categorical variables.

Abbreviations: CI, confidence interval; OR, odds ratio; SD, standard deviation.
